# Revisiting loxapine: a systematic review

**DOI:** 10.1186/s12991-015-0053-3

**Published:** 2015-04-01

**Authors:** Dina Popovic, Philippe Nuss, Eduard Vieta

**Affiliations:** Bipolar Disorders Program, Hospital Clínic, IDIBAPS, CIBERSAM, University of Barcelona, 170 Villarroel St., Barcelona, 08036, Catalonia Spain; Psychiatry and Medical Psychology Department, Hôpital Saint-Antoine, AP-HP, Paris, France and Sorbonne Universités-UPMC Univ Paris 06, UMR 7203, INSERM ERL 1157, CNRS LBM, Paris, France

**Keywords:** Loxapine, Antipsychotic, Agitation, Bipolar disorder, Schizophrenia

## Abstract

Loxapine is an antipsychotic used in psychiatry for over 40 years with a well-established profile. Loxapine is a dibenzoxazepine tricyclic antipsychotic agent, available for oral, intramuscular and inhalatory administration. In the light of the recent approval by the regulatory agencies of inhaled loxapine for use in the acute treatment of mild-to-moderate agitation in adults affected with schizophrenia or bipolar disorder, this article aims to critically review the available literature on loxapine, irrespective of its formulation.

This review examines the efficacy and tolerability of the various formulations of loxapine in the treatment of agitation and aggression in patients affected with schizophrenia, bipolar disorder and other psychiatric conditions.

A comprehensive and systematic literature search of PubMed/MEDLINE was conducted, and relevant pharmacodynamic and pharmacokinetic data was included. The findings from the literature were critically reviewed and synthesized.

The available data suggests that the antipsychotic efficacy of loxapine is similar to the efficacy of other typical or atypical antipsychotics, with an adverse effects profile comparable to that of the typical antipsychotics at high doses for chronic treatment. As an acute treatment in agitation associated with schizophrenia or bipolar disorder, inhaled loxapine was developed as an innovative and rapid option which appears to be efficacious and tolerable.

**Electronic supplementary material:**

The online version of this article (doi:10.1186/s12991-015-0053-3) contains supplementary material, which is available to authorized users.

## Introduction

Loxapine, an antipsychotic that has striking similarities to clozapine, has been recently re-launched as a treatment for agitation in schizophrenia and mania. Acute agitation, characterized by motor restlessness and mental tension, is a serious medical problem that may be present in various psychiatric disorders, including schizophrenia [1] and bipolar disorder [2] and can further escalate into aggressive behaviour [3]. It is important to identify agitation early in its course during its mild stages and achieve results quickly in order to prevent the escalation to aggressive or violent behaviour into more severe stages. In addition to the timely pharmacological treatment, acute interventions that target agitation usually also involve environmental and behavioural approaches [3]. Acute agitation requires prompt intervention to reduce the risk of patient injury and distress and to ensure the safety of other individuals (such as hospital staff, other patients, family members) [4].

The first attempts to treat agitation pharmacologically began with methylene blue, when Paul Erlich found that, when injected into frogs, it selectively stained nerve cells [5]. In 1899, Pietro Bodoni reported on its use to treat psychotic disturbances, in particular to calm psychotic agitation. In addition to morphine and scopolamine (hyoscine) combinations, barbiturates were main agents utilized to cure agitation until Delay and Deniker tested chlorpromazine. This compound was noted to be able to calm the agitation of patients with delirium, mania and psychosis even in monotherapy [5,6]. This observation was followed by numerous others, and the effectiveness of chlorpromazine in the treatment of agitated, overactive and manic states was widely confirmed [7,8].

Emergency sedation for behavioural disturbance in psychiatry in the mid-twentieth century, termed “rapid tranquillization”, received increasing attention after the arrival of antipsychotic drugs, which replaced older sedatives and became the agents most strongly associated with the treatment of aggression and challenging behaviour [9-11].

The mental health system was profoundly transformed by the use of antipsychotics. In the 1960s, the use of this class of medication was able to deliver effective treatment to outpatient clinics, community mental centres and mental hospitals. One of these compounds, loxapine, has been used for the treatment of schizophrenia since the mid-1970s [12,13] and is nowadays quite widely used in countries like France or Canada. In the light of the recent approval by the regulatory agencies of loxapine inhalation powder (ADASUVE®) in the USA and the EU for use in the acute treatment of agitation in adult patients with schizophrenia or bipolar disorder [4,14], this article aims to critically review the available literature on loxapine irrespective of its formulations.

## Methods

**Figure 1:**
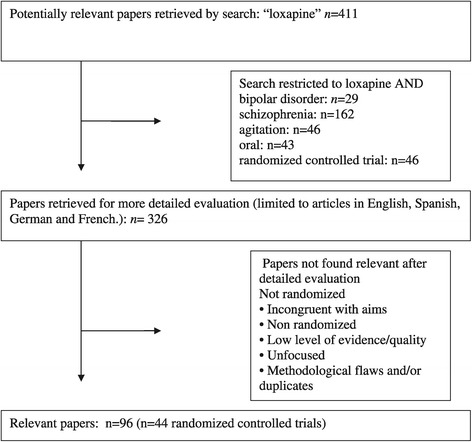
**Flow diagram of study design and results.**

A comprehensive and systematic literature search of all the articles on MEDLINE and PubMed, published up to March 2014 was performed, using the search term “loxapine” cross-referenced with “oral”, “bipolar disorder”, “schizophrenia”, “agitation” and/or “randomized controlled trial”. The search was supplemented by manually reviewing reference lists from the identified publications. The search included articles in English, Spanish, German and French. Relevant findings were then identified and synthesized in combination with additional literature regarding the pharmacodynamic and pharmacokinetic data. A general scheme of the output generated by the search is shown in Figure 1.

## Results

### Mechanism of action of loxapine

**Figure 2:**
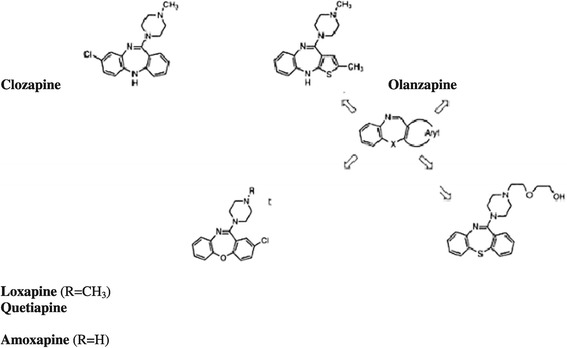
**Chemical structure of loxapine, amoxapine, clozapine, quetiapine and olanzapine.**

Loxapine is a dibenzoxazepine tricyclic antipsychotic agent, its chemical structure is similar to clozapine [13] (Figure 2) and is available for oral, intramuscular [13] and, following recent approval, inhalatory route [5].

The efficacy of loxapine is proposed to be mediated through high-affinity antagonism of postsynaptic dopamine D2 receptors and serotonin 5-HT2A receptors [15]. Although classified as a typical antipsychotic, loxapine has atypical characteristics. When antipsychotics are classified as typical or atypical, several considerations need to be kept in mind, including propensity for or lack of motor side effects such as extrapyramidal side effects and tardive dyskinesia and efficacy in treatment of negative symptoms.

The pharmacokinetic and pharmacodynamics properties of loxapine in favour of its atypical characteristics are the following:

- Receptor binding, especially at dopamine-2 (D2) serotonin-2A (5-HT2A) receptors and its high 5-HT2/D2 ratio, which is more characteristic of atypical antipsychotics [16-18]. Loxapine has a similar binding affinity as clozapine and olanzapine with a more potent 5-HT2A antagonism.
- Receptor occupancy: antipsychotic blockade of at least 60% of D2 R in the striatum is necessary for their therapeutic action; however, when 80% or more of D2 are blocked, EPS are likely to occur. Loxapine at 10–100 mg/day was found to be equipotent at blocking D2 and 5-HT2A receptors [19]. Positron emission tomography imaging revealed D2 receptor occupancy ranging from 43% to 90% and 5-HT2A receptor occupancy ranging from 27% to >98%; the loxapine dosage required to occupy 50% of D2 and 5-HT2A receptors was 9.6 and 13.6 mg/day, respectively [19]. Loxapine presents intermediate dissociation from the dopaminergic receptors, identical to olanzapine [20].
- Regarding affinity of other receptors, loxapine binds with higher affinity to D4 receptors than the other dopaminergic receptors, similarly to clozapine [13,21]. Additionally, loxapine has a higher affinity for D3 than D2 receptors [22].
- Antagonism of additional receptors: loxapine binds to noradrenergic, histaminergic H1 and cholinergic M1 receptors [13,23] (Table 1). Its interaction with these systems may influence the spectrum of its pharmacological effects that are associated with calming effects and suppression of aggressive behaviour.

**Table 1:** **Relative affinities of loxapine for various neurotransmitter receptors**

	**D1**	**D2**	**D3**	**D4**	**H1**	**M1**	**ά1**	**ά2**	**5-HT 1**	**5-HT 2**
Loxapine	++	+++	+++	+++	++	+	++	−	−	+++

+++ Very high, ++ high, + moderate, − low.

The pharmacokinetic characteristics of loxapine are listed in Table 2. Metabolism of loxapine includes demethylation to its primary N-demethylated metabolite amoxapine, a tricyclic antidepressant. The cytochrome P450 (CYP) enzyme CYP1A2 is involved in the hydroxylation of loxapine to 8-OH-loxapine, and CYP3A4 and CYP2D6 are involved in its hydroxylation to 7-OH-loxapine [10,13,24]. Loxapine also undergoes N-oxidation by flavonoid monoamine oxidases to form loxapine N-oxide and de-methylation by CYP3A4, CYP2C19 and CYP2C8 to form amoxapine. 8-OH-loxapine has no pharmacological activity at the D2 receptor, although 7-OH-loxapine (a minor metabolite) binds to D2 receptors with high affinity [10,13].

**Table 2:** **Pharmacokinetic parameters of loxapine**

**pKa**	**6.6**
Half-life	
Route oral	4 h (range 1–14)
Intramuscular	12 h (range 8–23)
Inhalatory route	4 h (range 6–8)
Time to peak concentration	
Route oral	1 h
Intramuscular	5 h
Inhalatory route	2 min (1–3)
Metabolites	7- and 8-Hydroxyloxapine
	Desmethyl-loxapine (amoxapine)
	7- and 8-Hydroxyamoxapine
	Ioxapine N-oxide
Excretion	Excretion of glucuronide conjugates in urine
	Unchanged in faeces

### Efficacy and tolerability of loxapine

The efficacy and tolerability of loxapine were well established through its large period of use not only in chronic and continuous treatment for schizophrenia or other psychiatric conditions [22] but also in acute psychotic stages.

#### The use of loxapine in schizophrenia

A 2007 Cochrane review included 41 studies, comparing loxapine to haloperidol, thiothixene, risperidone, clozapine and quetiapine [22]. As emerging from the review, compared with placebo, loxapine has an antipsychotic effect, based on the results of two randomized controlled trials (RCT), with a number needed to treat (NNT) of 3 [CI 3–5]. It is as effective as typical drugs in the short term (4–12 weeks) according to 13 RCTs. Very limited heterogeneous data suggest that, given intramuscularly (IM), loxapine may be at least as sedating as IM haloperidol and thiothixene [22].

According to data deriving from six RCTs, loxapine was also found as effective as atypicals (risperidone, quetiapine) and typicals such as perphenazine [25] and trifluoperazine [26] in small, head to-head trials [27]. The trial characteristics of the included trials are described in Additional file 1.

Regarding the tolerability of loxapine, common side effects of oral loxapine include parkinsonian-like symptoms such as tremor, hypomimia, rigidity, akathisia, drowsiness, dry mouth, constipation and weight changes [27].

According to a population-based cohort study of elderly people, the risk of death associated with conventional antipsychotic medications is comparable to and possibly greater than the risk of death associated with atypical antipsychotic medications [28]. In comparison with risperidone, haloperidol was associated with the greatest increase in mortality (mortality ratio 2.14, 95% CI 1.86–2.45) and loxapine, the lowest (mortality ratio 1.29, 95% CI 1.19–1.40) [29].

To date, no definitive association has been found between the use of antipsychotics during pregnancy and an increased risk of birth defects or other adverse outcomes. There are no studies concerning loxapine use in pregnancy in the literature, and the manufacturer reports outcomes from only three pregnancies with loxapine exposure: one child born with achondroplasia, one child born with multiple unspecified malformations and one child with tremors at 15 weeks of age [30]. However, the retrospective nature of these reports does not permit to reach conclusions regarding the safety of this drug in pregnancy. Overall, there is a paucity of information, with a lack of large, well-designed, prospective comparative studies on the safety of antipsychotics in pregnancy [30].

#### The use of loxapine in bipolar mania

Acute mania frequently constitutes a medical emergency, requiring prompt intervention to avoid destructive and possibly life-endangering behaviour [31]. Although it is evident that patients with acute bipolar mania require rapid and effective treatments that safely control this dangerous process [32], there is a paucity of empirical data regarding speed of action of antimanic treatments, an extremely important issue in clinical practice [33]. As shown in a recent meta-analysis, haloperidol has a faster onset of antimanic action than second-generation antipsychotics [33] although it also carries greater risk of switch into depression [34].

Chlorpromazine and haloperidol are the most studied typical antipsychotics in the treatment of bipolar mania [35]. One small placebo-controlled study supports the efficacy of chlorpromazine [36] while several studies support the efficacy of haloperidol in this indication. The first publications date back to 1975 [24], while more recent studies support the efficacy of up to 30 mg/day of haloperidol for the treatment of acute mania, with a response rate vs. placebo at 3 weeks of 47–56.1% vs. 20–35%, respectively [37-41]. To date, there have been no trials assessing the efficacy of the oral or intramuscular formulation of loxapine in acute treatment of manic episode. One RCT found inhaled loxapine effective in the reduction of agitation as early as 10 min after first administration in bipolar patients in manic or mixed episode [42]. Typical antipsychotics such as haloperidol, zuclopenthixol or loxapine are still widely prescribed in the treatment of mania, due to the need to acquire adequate and rapid sedation or even the so-called chemical containment [43]. However, the treatment with typical antipsychotics carries a potential risk and associated side effects such as extrapyramidal side effects, depressogenic effect and malignant syndrome [43]. It is noteworthy that some authors have established that the patients affected with mood disorders are more prone to develop extrapyramidal side effects than the schizophrenic patients [44,45], which may contribute to limit the prescription of typical antipsychotics in this population beyond punctual administration.

#### Loxapine in the treatment of agitation across psychiatric disorders

Timely treatment of agitation is essential, also considering that it can further escalate into aggressive behaviour. Aggressive behaviour is variable in intensity and targets with a wide range of expression either verbal, against objects, self or other persons (frequently called violence) [3].

Reports evaluating loxapine’s effects date back to the 1970s. Five small-scale, randomized, double-blind trials studies assessed the relationship between loxapine treatment and aggression [46-50]. Whatsoever, the small sample sizes (from 30 to 54 patients) in all of these studies limit the generalisability of the findings.

Although data supports the use of haloperidol as an efficacious treatment of aggression in autistic disorder [51-54], the empirical data is less clear regarding the utility of the other typical antipsychotics in this patient population. Only anecdotic reports suggest anti-aggressive effects of loxapine [55] and amoxapine in autistic children [56], but there is a clear need for additional research in autistic and other psychiatric populations.

Recently, inhaled loxapine (ADASUVE) has been approved by the Food and Drug Administration and European Medical Agency for the acute treatment of agitation associated with schizophrenia or bipolar disorder in adults. The approval was based on one phase II and two phase III efficacy trials in the treatment of acute agitation in the above-mentioned disorders [42,57,58]. In two phase III studies (one in subjects with schizophrenia, the other in subjects with bipolar disorder) inhaled loxapine doses of 5 and 10 mg were both superior to placebo 10 min after administration. Pooled data from these three efficacy studies suggest a NNT for response of 4 (95% CI 3–5) and 3 (95% CI 3–4) for inhaled loxapine (5 or 10 mg vs. placebo) for agitation associated with schizophrenia or bipolar disorder, respectively. These values are in the range with effect size observed for intramuscular administration of other antipsychotics [59].

According to data pooled from the three existing efficacy studies of inhaled loxapine, no clinically relevant extrapyramidal side effects or akathisia have emerged following administration of loxapine 10 mg or 5 mg, and the most commonly encountered adverse event was short-term dysgeusia, with a number needed to harm vs. placebo of 10 (95% CI 7–22) or 12 (95% CI 8–26), respectively [59]. Given that the route of administration is inhalation, active airways disease (asthma and chronic obstructive pulmonary disease) are contraindicated due to the risk of bronchospasm [27]. There is no apparent QT prolongation associated with the therapeutic dose of inhaled loxapine [60].

## Discussion

The present review of the available literature derives from the large experience of 40 years of loxapine use in chronic treatment of psychiatric disorders and their agitated stages.

The systematic Cochrane review assessing all randomized controlled trials that compared loxapine (in dosages up to 300 mg/day) to other chronic treatments in schizophrenia concluded that loxapine was not distinct from typical or atypical drugs in terms of its effects on global or mental state and exhibited a similar adverse effect profile to typical antipsychotics but may cause more extrapyramidal adverse effects when compared with atypical antipsychotics [22].

Furthermore, loxapine is more effective than other ‘typical’ antipsychotics in reducing the negative symptoms of schizophrenia [57]. Extrapyramidal side effects are not usually observed at clinically effective antipsychotic doses [61]. Loxapine, although classified as a conventional antipsychotic, at low doses (<50 mg/day) may be considered as an atypical one [13,18].

Considering the above-mentioned data, and with the aim of improving the treatment of agitated patients with schizophrenia and bipolar disorder [62], inhaled loxapine was developed and approved as an effective and tolerable option. This innovative approach could be a good option in the management of agitation, intercepting the escalation to its severe stages.

Existing evidence supports the efficacy of clozapine in reducing suicidal behaviour in patients with schizophrenia [63-68]. In view of the similarities between clozapine and loxapine, future studies should investigate potential anti-suicidal properties of loxapine.

## Conclusions

Data suggests that loxapine is an antipsychotic drug with an efficacy similar to other typical or atypical agents in terms of antipsychotic efficacy and adverse effects profile similar to that of the typical antipsychotics at high doses for the chronic treatment. As an acute treatment of agitation associated with schizophrenia or bipolar disorder, inhaled loxapine, as delivered through a fast-heating device (Staccato), was developed as an innovative and particularly rapid pharmacological option which appears to be efficacious and tolerable in most clinical settings where psychiatric agitation is problematic.

## Additional file

Additional file 1:
**Characteristics of the included trials** **[** 69 **-** 102 **].**
