# Supplementary material for: Revisiting loxapine: a systematic review
Source: Ann Gen Psychiatry. 2015 Apr 1;14:15. doi: 10.1186/s12991-015-0053-3 (PMC4391595; doi:10.1186/s12991-015-0053-3)
Supplement: Additional file 1: — Characteristics of the included trials [ 69 - 102 ]. [file 12991_2015_53_MOESM1_ESM.doc]

**Additional file 1** **Characteristics of the included trials**

| **Trial** | **Intervention** | **Participants** | **Trial characteristics** | **Outcome measures** |
| --- | --- | --- | --- | --- |
| Allen 2011 [58] | 1. Inhaled loxapine dose, 5 mg single inhalation | Diagnosis: agitation in schizophrenia or schizoaffective disorder (DSM-IV criteria) | Allocation: randomized, double blind, placebo-controlled study | • PANSS-EC (2 h following treatment) |
|  | *N* = 45 |  |  | • CGI-I |
|  | 2. Inhaled loxapine dose, 10 mg single inhalation |  |  | • Behavioural activity rating scale (BARS) |
|  |  |  | Setting: clinical or hospital setting |  |
|  |  |  | Duration: single inhalation | • Time to first rescue medication |
|  |  | *N* = 129 |  |  |
|  | *N* = 41 | Age 18–65 years |  |  |
|  | 3. Inhaled placebo (using the Staccato system), single inhalation | History: inpatients/outpatients/emergency room |  |  |
|  | *N* = 43 |  |  |  |
| Bagadia 1980 [69] | 1. Loxapine, 10 mg/day increased to 120 mg/day | Diagnosis: schizophrenia with onset between 13 and 19 years (ICD-10) | Allocation: randomized, double-blind identical capsules | • Leaving the study early |
|  |  |  |  | • Drug preference |
|  |  |  |  | • Patients self-evaluation |
|  |  | *N* = 55 |  |  |
|  |  | Age: mean ~17 years, range 14–24 | Duration 4 weeks |  |
|  |  |  |  | • Adverse effects |
|  |  |  |  | • CGI (no SD) |
|  |  | History: outpatients |  |  |
|  |  |  |  | • BPRS (no SD) |
|  |  |  |  | • NOSIE (no SD) |
|  | *N* = 25 |  |  |  |
|  | 2. Trifluperazine dose, 2.5 mg/day increased up to 25 mg/day max. *N* = 30 |  |  |  |
| Bishop 1970 [70] | 1. Loxapine, 20 mg/day, increased to 120 mg/day maximum | Diagnosis: schizophrenia, chronic | Allocation: randomized | • CGI |
|  |  |  |  | • Adverse effects |
|  |  |  | Double-blind |  |
|  |  | *N* = 24 | Duration 8 weeks—preceded by 4 weeks washout + 2-week assessment period |  |
|  |  | Age: mean ~ 44 years, range 30–55 |  |  |
|  | *N* = 12 |  |  |  |
|  | 2. Trifluoperazine, 10 mg/day, increased to 60 mg/day maximum. *N* = 12 |  |  |  |
|  |  | Sex, 12 males, 12 females |  |  |
|  |  | History: inpatients ~ 17 years, range 5–29 years |  |  |
| Charalampous 1974 [71] | 1. Loxapine dose range, 50–150 mg/day, mean 147.5 mg | Diagnosis: schizophrenia (by two psychiatrists) | Allocation: randomized | • Adverse effects (TESS, use of anticholinergic drugs) |
|  |  |  | Double-blind (identical capsules) |  |
|  |  | *N* = 60 |  |  |
|  | *N* = 20 | Age: mean ~ 26 years, range 18–53 |  | • BPRS (no SD) |
|  |  |  | Duration 4 weeks preceded by 1 week washout |  |
|  | 2. Thiothixene dose, range 20–60 mg/day, mean 51.9 mg |  |  |  |
|  |  | Sex, 58 males, 2 females | Setting: single centre |  |
|  |  | History: inpatients, ill <6 years, healthy; mean length ill ~2.5 years |  |  |
|  | *N* = 20 |  |  |  |
|  | 3. Placebo |  |  |  |
|  | *N* = 19 |  |  |  |
|  | Chloral hydrate and trihexyphenidyl as required |  |  |  |
| Clark 1972 [72] | 1. Loxapine dose, 10 mg/day increased to 100 mg/day in 25 days. *N* = 18 | Diagnosis: schizophrenia, confirmed by project psychiatrist | Allocation: random, stratified age and sex | • CGI-I |
|  |  |  |  | • Adverse effects (physical examination, lab results, ECG and eye examination) |
|  |  |  | Double blind-identical capsules |  |
|  | 2. Chlorpromazine dose, 100 mg/day increased to 1gm/day. *N* = 19 | *N* = 55 |  |  |
|  |  |  |  | • CGI (severity) (no SD) |
|  |  | Age, 21–60 years |  |  |
|  |  |  |  | • BPRS (no SD) |
|  |  | Sex, 31 males, 24 females | Duration 12 weeks, with 12 weeks washout | • NOSIE (no SD) |
|  |  | History: inpatients, ill for at least 2 years |  |  |
|  | 3. Placebo. *N* = 18 |  |  |  |
|  | Antiparkinsonian medication allowed as required |  |  |  |
| Clark 1975 [73] | 1. Loxapine dose, 100 mg/day, mean 71 mg/day. *N* = 15 | Diagnosis: schizophrenia, confirmed by research psychiatrists | Allocation: random—pre-randomized list, blocks of 3, provided by drug company | • CGI-I, CGI-S, use of additional sedation |
|  | 2. Trifluoperazine dose, 50 mg/day, mean 36 mg/day. *N* = 14 |  |  | • Adverse effects |
|  |  | *N* = 42 |  | • Leaving the study early |
|  |  |  | Double-blind identical capsules |  |
|  |  |  |  | • Laboratory tests |
|  |  | Age: range 21–57 years | Duration 4 weeks | • Physiological measures (ECG, weight) |
|  | 3. Placebo. *N* = 13 |  |  |  |
|  | Short-acting sedatives and antiparkinsonian medication as required |  | Setting: single centre |  |
|  |  | Sex, 21 males, 16 females, 6 unreported |  | • BPRS (no SD) |
|  |  | History: ill > 2 years, healthy, not able to bear children |  |  |
| Clark 1977 [74] | 1. Loxapine dose, 100 mg/day. *N* = 12 | Diagnosis: schizophrenia (DSM-II) | Allocation: random—pre-randomized list, blocks of 3, provided by drug company | • CGI-I, CGI-S, use of additional sedation |
|  | 2. Loxapine dose, 50 mg/day. *N* = 13 | *N* = 38 |  | • Adverse effects |
|  |  | Age: range 21–57 years |  | • .Leaving the study early |
|  | 3. Placebo. *N* = 13 |  |  |  |
|  |  |  | Double-blind identical capsules |  |
|  |  |  |  | • Laboratory tests |
|  |  | Sex, 11 males, 27 females |  |  |
|  |  |  |  | • Physiological measures (ECG, weight) |
|  |  |  | Duration 12 weeks—“..effects of previous treatment allowed to dissipate over a period of 12 weeks” before trial |  |
|  |  | History: >2 years ill and institutionalizations without remission, healthy, not pregnant, inpatients |  | • BPRS (no SD) |
|  | Short-acting sedatives and antiparkinsonian medication as required |  |  |  |
|  |  |  | Setting: single centre |  |
| Du 2003 [75] | 1. Loxapine dose, range 68–305 mg. *N* = 30 | Diagnosis: schizophrenia (CCMD-3) | Allocation: randomized | • Adverse effects: TESS |
|  |  |  | Blinding: none | • Laboratory tests |
|  |  |  | Duration 8 weeks | • Physiological measures: ECG, EEG |
|  | 2. Risperidone dose, range 1–6 mg. *N* = 30 | *N* = 60 |  |  |
|  |  | Loxapine group 18 males, 12 female; average age 29 | Setting: single centre |  |
|  |  | Risperidone group 17 males, 13 females; average age 26 |  |  |
|  |  | History: hospitalized patients |  |  |
| Dube 1976 [76] | 1. Loxapine dose, 20–80 mg/day, mean 34.3 mg/day. *N* = 26 | Diagnosis: schizophrenia. *N* = 52 | Allocation: randomized double-blind identical capsules | • CGI |
|  |  |  |  | • Adverse effects |
|  |  |  |  | • Leaving the study early |
|  |  | Age: mean ~ 32 years, range 18–55 |  |  |
|  | 2. Chlorpromazine dose, 200–800 mg/day, mean 320 mg/day. *N* = 26 |  |  |  |
|  |  |  |  | • BPRS (no SD) |
|  |  |  | Duration 12 weeks |  |
|  |  | Sex 52 males |  |  |
|  |  | History: <2 years ill, healthy, no co-existing mental illnesses | Setting: single centre |  |
| Dubin 1996  [77] | 1. Loxapine dose, mean 75.5 mg/day IM, range 25–175 mg/day IM. *N* = 30 | Diagnosis: schizophrenia (52), bipolar manic (9) (DSM-III) | Allocation: randomized—randomization table | • Global effect (sedation, requiring further injections) |
|  |  |  |  | • Dropped from study |
|  | 2. Thiothixene dose, mean 31 mg/day IM, range 20–60 mg/day IM, *N* = 31 |  | Double-blind identical ampoules, staff administering medication not blinded, assessors blind |  |
|  |  | *N* = 61 |  |  |
|  |  | Age: mean ~ 35 years, range 18–65 |  | • BPRS (no SD). |
|  |  |  |  | • Side effects: (only data for 5 day oral phase available) |
|  |  | Sex ~27 males, ~31 females |  |  |
|  | IM for first 24 h, then oral. IM injections every 30 min as needed, until BPRS reduced or sedation occurred. Chloral hydrate, trihexyphenidyl/ benztropine as required | Inclusion: BPRS score of 6/7 in >2 pre-specified symptom categories. | Duration 6 days—preceded by 24 h washout (only data from first 72 h used) |  |
|  |  | History: admitted as psychiatric emergency, healthy, drug sensitivity, not pregnant or lactating, no coexisting mental illness |  |  |
|  |  |  | Setting: single centre |  |
| Fruensgaard 1977 [46] | 1. Loxapine dose, 25–50 mg/6–12 h IM, mean 130 mg/day IM. *N* = 15 | Diagnosis: acute schizophrenia (12), psychogenic psychosis (18) | Allocation: randomized | • CGI, sedation |
|  |  |  |  | • Adverse effects: pain at injection site |
|  |  |  | Double-blind identical ampoules |  |
|  |  | *N* = 30 |  | • BPRS (no SD) |
|  |  | Age: mean ~ 40 years, range 19–65 | Duration 72 h—preceded by 12 h washout (study continued for 4 weeks but not reported) |  |
|  | 2. Thiothixene dose, 2.5–5 mg/6–12 h IM, mean 12 mg/day IM. *N* = 15 |  |  |  |
|  |  | Sex, 7 males, 23 females. |  |  |
|  |  | Inclusion: healthy, not pregnant, no coexisting mental illness, mania or treatment with ECT in last 8 weeks |  |  |
|  |  |  | Setting: single centre |  |
|  | Both given with biperiden. IM for first 24 h, then oral. IM injections every 30 minutes as needed, until BPRS reduced or sedation occurred |  |  |  |
|  |  | History: duration of present episode: <1 week (14), 1 week–1 month (13), >1 month (3) |  |  |
| Fruensgaard 1978 [25] | 1. Loxapine dose, 10 mg bid increased to 150 mg/day. *N* = 23 | Diagnosis: acute schizophrenia (7), acute schizophreniform psychotic episodes or acute exacerbations of a chronic schizophrenic process, psychogenic (reactive) psychosis (15), chronic schizophrenia (25) | Allocation: randomized double blind | • CGI |
|  |  |  |  | • Adverse effects |
|  |  |  |  | • Physiological effects, laboratory tests, ECG |
|  |  |  | Duration: group one—3 weeks, group two—12 weeks |  |
|  |  |  | Setting: multicenter |  |
|  |  | *N* = 47 |  |  |
|  |  | Age: range 16–67 years |  |  |
|  |  | Sex 32 males, 15 females |  |  |
|  | 2. Perphenazine dose, 8 mg bid increased to 120 mg/day. *N* = 24 |  |  |  |
|  | Chloralodolol used for insomnia and antiparkinsonian medications used as required |  |  |  |
| Gallant 1971 [78] | 1. Loxapine dose, 20 mg/day increased to 120 mg/day. *N* = 12 | Diagnosis: chronic schizophrenia | Allocation: randomized by random numbers | • Adverse events |
|  |  | *N* = 24 |  |  |
|  |  |  | Double blind |  |
|  |  |  | Duration: 8 weeks, with 4 weeks washout |  |
|  | 2. Trifluperazine dose, 10 mg/day increased up to 60 mg/day. *N* = 12 | Age: range 30–55. |  |  |
|  |  | Sex, 11 males, 13 females |  |  |
|  |  | Design: phase 2 trial |  |  |
|  | Antiparkinsonian medication given as required. |  |  |  |
| Huang 1997 [79] | 1. Loxapine dose, range: 50–300 mg. *N* = 104 | Diagnosis: schizophrenia | Allocation: randomized | • BPRS |
|  |  |  |  | • CGI |
|  |  | *N* = 205 | Double blind | • Adverse effects: TESS |
|  |  |  | Duration 8 weeks |  |
|  |  |  |  | • Physiological effects: EEG |
|  | 2. Chlorpromazine dose range: 75–600 mg | Age: mean ~35 years, range 18–60 |  |  |
|  |  |  | Setting: multicenter |  |
|  |  | Sex: 123 males, 82 females |  |  |
|  | *N* = 101 | History: hospitalized |  |  |
| Kiloh 1976 [80] | 1. Loxapine dose, 10 mg/day increased progressively—acute group mean ~37 mg/day (SD ~22), chronic group 56 mg/day (SD 20). *N* = 30 | Diagnosis: schizophrenia (Slater and Roth criteria) | Allocation: randomized - by a prearranged system | • CGI |
|  |  |  | Double blind |  |
|  |  | *N* = 57 |  |  |
|  |  | Age: less than 69 | Duration: 12 weeks with a 2 weeks washout |  |
|  | 2. Trifluperazine dose, 5 mg/day increased progressively—acute group mean ~24 mg/day (SD 14.5), chronic group 31 mg/day (SD 11.7). *N* = 27 |  |  |  |
|  |  | Sex: no details available |  |  |
|  |  | History: inpatients, duration ill—acute <2 years, chronic >2 years  Note: Data extracted clubbing both acute and chronic patients |  |  |
|  | Diazepam, barbiturates and benztropine for adverse effects as required |  |  |  |
|  |  |  |  | • Adverse effects - physical examination, ophthalmic examination, laboratory tests, ECG |
| Kramer 1978 [81] | 1. Loxapine dose, mean 74 mg/day. *N* = 34 | Diagnosis: schizophrenia, acute (DSM-II) | Allocation: randomized—no further details | • Leaving the study early. Dropped from analysis |
|  |  |  |  | • CGI (>50% attrition) |
|  | 2. Thioridazine dose mean, 442 mg/day. *N* = 35. | *N* = 69. | double-blind identical ampoules |  |
|  |  |  |  | • BPRS (>50% attrition) |
|  |  | Age: mean ~31 years, range >18–57 |  |  |
|  |  |  |  | • NOSIE (>50% attrition) |
|  |  |  | Duration 4 weeks—preceded by 2-week drug-free period |  |
|  | Doses individually titrated, antiparkinsonian medication as required |  |  | • Side effects: DOTES (>50% attrition) |
|  |  | Sex, 21 males, 35 females, 13 not reported |  |  |
|  |  |  |  | • Efficacy: (analysis of covariance) |
|  |  | Exclusion: ill health, < 1 week of study medications |  |  |
|  |  |  | Setting: single centre | • Physiological measures: ECG, hand writing (>50% attrition) |
|  |  | Note: loss to follow up 60% |  |  |
|  |  | Only data from the outcome of ‘leaving the study early’ used |  |  |
| Kwentus 2012 [42] | 1. Inhaled loxapine 5 mg. *N* = 105 | Diagnosis: agitation in patients with bipolar I disorder-manic or mixed episodes (DSM IV) | Allocation: randomized, double blind, placebo-controlled, parallel group inpatient study | Changes from baseline in the PANSS-EC from 10 min through 24 h after dose 1 |
|  | 2. Inhaled loxapine 5 mg. *N* = 104 |  |  |  |
|  | 3. Inhaled placebo (using the Staccato system) *N* = 105 |  |  |  |
|  |  |  | Duration: single dose administered and patients evaluated for 24 h |  |
|  | If required, up to two additional doses of study drug and⁄or lorazepam rescue medication | *N* = 314 |  | • CGI-I (2 h after dose 1) |
|  |  |  |  | • Safety: adverse events, vital signs, physical examinations and laboratory tests |
|  |  |  | Setting: multicenter (17 centres) |  |
| Lesem 2011 [57] | 1. Inhaled loxapine 10 mg (1–3 inhalations) *N* = 110 | Diagnosis: acute agitation in schizophrenia (DSM-IV) *N* = 344 | Allocation: randomized double-blind, placebo-controlled, parallel-group study | • PANSS-EC (2 h after dose one) |
|  |  |  |  | • CGI-I (2 h after dose one) |
|  | 2. Inhaled loxapine 5 mg |  |  |  |
|  |  | Sex, males and females, 18 to 65 years old |  |  |
|  |  |  | Duration 1–3 inhalations |  |
|  | (1–3 inhalations) |  | Setting: inpatients or outpatients or emergency room |  |
|  | *N* = 114 |  |  |  |
|  | 3. Inhaled placebo |  |  |  |
|  | (1–3 inhalations) |  |  |  |
|  | *N* = 114 |  |  |  |
|  | Lorazepam rescue was permitted after dose two |  |  |  |
| Li 2004 [82] | 1. Loxapine mean dose, 208 mg/day (max 306 mg/day). *N* = 30 | Diagnosis: schizophrenia (CCMD-3) | Allocation: randomized | • PANSS |
|  |  |  | Blinding: non-blind |  |
|  |  | *N* = 60 |  |  |
|  | 2. Clozapine mean dose, 415 mg/day (max 600 mg/day). *N* = 30 | Age: mean ~30, range 18–60 | Duration 8 weeks |  |
|  |  | Sex, 38 males, 22 females |  |  |
|  |  | History: hospitalized |  |  |
| Li 2005a [83] | 1. Loxapine: dose, no average dose, max dose 272 mg/day. *N* = 44 | Diagnosis: schizophrenia (CCMD3) | Allocation: randomized | Mental state: PANSS |
|  |  |  | Blinding: not mentioned | Adverse effects: EPSE, abnormal ECG, agitation, insomnia |
|  |  | *N* = 87 |  |  |
|  | 2. Risperidone: dose, no average dose, max dose 6 mg/day. *N* = 43 | Age: mean ~33 | Duration 8 weeks |  |
|  |  | Sex: not reported |  |  |
|  |  | History: duration ill ~ 5 years |  | Note: 1 dropout from the loxapine group after 1 week due to difficulty swallowing and hypermyotonia |
| Li 2005b [84] | 1. Loxapine: dose range 34–340 mg. *N* = 34 | Diagnosis: schizophrenia (CCMD-3) | Allocation: randomized | • PANSS |
|  |  |  |  | • TESS |
|  |  |  | Blinding: not mentioned | Physiological measures: EEG, ECG |
|  | 2. Risperidone: dose range 1–7 mg. *N* = 34 | *N* = 68 |  |  |
|  |  | Age: mean ~25, range 16–50 | Duration 8 weeks |  |
|  |  | Sex, 40 males, 28 females | Setting: single centre |  |
|  |  | History: hospitalized |  |  |
| Liu 2005 [85] | 1. Loxapine: dose 68–204 mg/day. *N* = 40 | Diagnosis: schizophrenia (CCMD-3) | Allocation: randomized  Blinding: not mentioned | • PANSS |
|  |  |  |  | • TESS |
|  | 2. Chlorpromazine: dose 250–600 mg/day. *N* = 40 | *N* = 80 |  |  |
|  |  | Age: mean ~28 years | Duration 8 weeks |  |
|  |  | Sex, 43 males, 37 females | Setting: single centre |  |
|  |  | History: mean duration ill ~ 22 months |  |  |
| Lu 2003 [86] | 1. Loxapine: dose range 34–272 mg. *N* = 62 | Diagnosis: schizophrenia (CCMD-3) | Allocation: randomized | • PANSS |
|  |  |  |  | • TESS |
|  |  |  | Blinding: non-blind | Laboratory tests: bloods, urine, EEG |
|  | 2. Clozapine: dose range 25–600 mg. *N* = 60 | *N* = 122 |  |  |
|  |  | Age: mean ~34 years, range 16–56 | Duration 6 weeks |  |
|  |  | Sex, 81 males, 41 females |  |  |
|  |  | History: hospitalized |  |  |
| Malik 1980 [87] | 1. Loxapine: dose mean 91.5 mg/day. *N* = 27 | Diagnosis: schizophrenia | Allocation: randomized | • CGI |
|  |  |  |  | • Adverse effects |
|  |  |  |  | • Drug preference |
|  |  | *N* = 54 | Blinding: double—identical capsules | • Dropped from analysis |
|  | 2. Trifluoperazine: dose mean 23.57 mg/day. *N* = 27 | Age: mean ~17 years, range >14–19 |  |  |
|  |  |  |  | • BPRS (no SD) |
|  |  |  | Duration 28 days |  |
|  | Antiparkinsonian medication as required | Sex, 25 males, 27 females, 2 not reported |  |  |
|  |  |  | Setting: single centre |  |
|  |  | Exclusion: sensitivity to study drugs, ECT in last 8 weeks, co-existing mental illness, ill health |  |  |
| Moore 1975 [88] | 1. Loxapine: dose 20 mg–120 mg/day. *N* = 29 | Diagnosis: schizophrenia, acute or exacerbations of chronic | Allocation: no details | • CGI |
|  |  |  |  | • Use of additional sedation |
|  |  |  | Blinding: double - identical capsules |  |
|  | 2. Chlorpromazine: dose 200–1200 mg/day. *N* = 29 |  |  | • Adverse effects: TESS, use of antiparkinsonian drugs |
|  |  |  | Duration 6 weeks—preceded by 2 week washout |  |
|  |  | *N* = 54 |  |  |
|  | Antiparkinsonian or sedative medication as required. | Age: mean ~ 37 years |  | • Dropped from analysis |
|  |  |  | Setting: single centre | • Laboratory tests |
|  |  | Sex, 25 males, 27 females, 2 not reported |  |  |
|  |  | History: hospitalized |  |  |
| Moyano 1975 [47] | 1. Loxapine: dose 20–120 mg/day. *N* = 25 | Diagnosis: schizophrenia, chronic *N* = 49 | Allocation: no details | • Adverse effects: TESS |
|  |  |  | Blinding: double—identical capsules | • Dropped from analysis |
|  | 2. Trifluoperazine: dose 20 mg–40 mg/day. *N* = 24 |  |  |  |
|  |  |  |  | • Physiological measures (ophthalmic tests) |
|  |  | Age: mean ~ 47 years, all >21 |  |  |
|  |  |  | Duration 12 weeks—preceded by 4-week washout |  |
|  |  |  |  | • Laboratory tests |
|  |  | Sex: 30 males, 19 females |  |  |
|  | Antiparkinsonian or sedative medication as required | History: prolonged drug treatment, hospitalized patients |  |  |
|  |  |  | Setting: single centre |  |
|  |  | Exclusion: co-existing mental illness, ill health, <4 weeks of study medication |  |  |
| Pool 1976 [89] | 1. Loxapine: dose 10 mg/day increased to 200 mg/day, mean 87.5 mg/day. *N* = 25 | Diagnosis: schizophrenia confirmed by two psychiatrists (no other details) | Allocation: random (pre-arranged procedure) | • CGI |
|  |  |  | Blinding: double-blind, identical capsules in bottles which were numbered only with the person’s study number |  |
|  | 2. Haloperidol: dose 2 mg/day increased to 16 mg/day, mean 9.8 mg/day. *N* = 25 | *N* = 75 |  |  |
|  |  | Age 13–18 years |  |  |
|  |  | Sex, 43 males, 32 females |  |  |
|  |  | History: inpatients |  |  |
|  | 3. Placebo. *N* = 25 |  |  |  |
|  | Antiparkinsonian medications, sodium amobarbital used as required |  |  |  |
|  |  |  | Duration: 4 weeks, with 5 day washout |  |
| Rifkin 1984 [90] | 1. Loxapine: dose mean 128.6 mg/day (SD 38). *N* = 31 | Diagnosis: schizophrenia, paranoid (RDC) | Allocation: randomized | • CGI |
|  |  |  |  | • Leaving the study early |
|  |  |  |  |  |
|  | 2. Chlorpromazine: dose mean 1288 mg/day (SD 358). *N* = 33 | *N* = 64 | Blinding: double- identical capsules, evaluation by psychiatrist blind to drug taken |  |
|  |  |  | Duration 4 weeks |  |
|  |  |  | Setting: single centre |  |
|  |  | Age 18–60 years |  |  |
|  |  | Sex 41 males, 23 females |  |  |
|  |  | History: hospitalized |  |  |
|  |  |  |  |  |
|  |  | Exclusion: pregnant or risk of co-existing mental illnesses, ill health, recent amphetamine abuse, hospitalized patients |  |  |
|  | Antiparkinsonian or benzodiazepine as required |  |  |  |
| Schiele 1975 [12] | 1. Loxapine: dose mean 110 mg/day. *N* = 26 | Diagnosis: schizophrenia, chronic | Allocation: random | • CGI |
|  |  |  |  | • Adverse effects (use of antiparkinsonian drugs) |
|  |  |  | Blinding: double—identical opaque capsules |  |
|  | 2. Chlorpromazine: dose mean 1100 mg/day. *N* = 24 | *N* = 64 |  |  |
|  |  | Age: mean ~45 years, range 25–74 |  |  |
|  |  |  | Duration 12 weeks—preceded by 1 week placebo period |  |
|  |  |  |  | • Leaving the study early |
|  | Antiparkinsonian or benzodiazepine as required |  |  | • Laboratory tests |
|  |  | Sex 50 males, 14 females | Setting: single centre |  |
|  |  | History: long-term hospitalization |  |  |
|  |  | Exclusion: ill health |  |  |
| Selman 1976 [49] | 1. Loxapine: dose 50–150 mg/day. *N* = 29 | Diagnosis: schizophrenia, acute or exacerbations of chronic (by two psychiatrists) | Allocation: random | • CGI. |
|  |  |  |  | • Adverse effects |
|  |  |  | Blinding: double—identical capsules | • Leaving the study early |
|  | 2. Haloperidol: dose 4–12 mg/day. *N* = 29 |  |  |  |
|  |  |  |  | • Dropped from analysis |
|  |  |  | Duration: 12 weeks—preceded by a 2-week placebo period |  |
|  | 3. Placebo. *N* = 29 | *N* = 87 |  |  |
|  | Antiparkinsonian, chloral hydrate or paraldehyde as required | Age: mean ~ 32 years |  |  |
|  |  | Sex, 69 males, 18 females | Setting: single centre |  |
|  |  | History: long-term hospitalization |  |  |
|  |  | Exclusion: ill health, < 4 weeks of study medication |  |  |
| Seth 1979 [91] | 1. Loxapine: dose 20–90 mg/day. *N* = 36 | Diagnosis: schizophrenia, chronic (by two psychiatrists) | Allocation: random | • Leaving the study early |
|  |  |  | Blinding: double—identical capsules | • Dropped from analysis |
|  | 2. Trifluoperazine: dose 5–45 mg/day. *N* = 36 |  |  |  |
|  |  | *N* = 72 |  | • Adverse effects |
|  |  | Age: mean ~ 30 years, range <20–49 | Duration 12 weeks—preceded by 4 week washout period |  |
|  |  | Sex, 28 males, 36 females, 8 not reported |  |  |
|  |  |  | Setting: single centre |  |
|  |  | History: hospitalized patients |  |  |
|  |  | Exclusion: ill health, pregnant or risk of, substance abuse |  |  |
| Shopsin 1972 [92] | 1. Loxapine: dose 30–120 mg/day. *N* = 15 | Diagnosis: schizophrenia, acute (SPS), undertaken by two psychiatrists | Allocation: random | • Global effect (discharge) |
|  |  |  |  | • Leaving the study early |
|  |  |  | Blinding: double—identical capsules, rated by independant psychologists |  |
|  | 2. Chlorpromazine: dose 300–1200 mg/day. *N* = 15 |  |  |  |
|  |  |  |  | • TESS |
|  |  | *N* = 30 |  |  |
|  |  |  | Duration 3 weeks—preceded by 7 day placebo washout period |  |
|  |  |  | Setting: single centre |  |
|  | Antiparkinsonian drugs, chloral hydrate and paraldehyde as required | Age range 21–62 years |  |  |
|  |  | Sex: male and female (some participants not reported on) |  |  |
|  |  | History: newly hospitalized |  |  |
|  |  | Exclusion: ill health, pregnant or risk of, substance abuse, unmanageable behaviour, refusal to take oral medication, spontaneous remission during placebo phase |  |  |
|  |  | Inclusion: demonstrating disturbance of affect and association |  |  |
| Simpson 1976  [26] | 1. Loxapine: dose mean 74 mg/day, range 30–120 mg/day. *N* = 24 | Diagnosis: schizophrenia, acute (no further details) | Allocation: randomized | • Global effect (discharge) |
|  |  |  |  | • Leaving the study early |
|  |  |  | Blinding: double—identical capsules |  |
|  |  |  |  | • Adverse effects (neurological rating scale, unwanted effects checklist) |
|  | 2. Trifluoperazine: dose mean 35 mg/day, range 20–50 mg/day. *N* = 19 | *N* = 43 |  |  |
|  |  | Age: mean 32 years, range 16–61 | Duration 4 weeks—preceded by 3-day drug-free period, evaluation carried out by the same physician, study lasted over 3 years |  |
|  |  | Sex, 27 males, 16 females |  |  |
|  | Antiparkinsonian drugs and chloral hydrate as required. | Exclusion: ill health |  |  |
|  |  | History*:* newly hospitalized |  |  |
|  |  |  | Setting: single centre |  |
| Steinbook 1973 [93] | 1. Loxapine: dose range 30–150 mg/day. *N* = 26 | Diagnosis: schizophrenia, acute (no further details) | Allocation: randomized | • Adverse effects (use of antiparkinsonian drugs) |
|  |  |  | Blinding: double—identical capsules |  |
|  | 2. Chlorpromazine: dose range 10–1200 mg/day. *N* = 28 |  |  |  |
|  |  | *N* = 54 |  |  |
|  |  | Age: mean ~34 years, range 21–65 | Duration 6 weeks—preceded by 3 day drug-free period |  |
|  | Antiparkinsonian medication as required |  |  |  |
|  |  |  |  |  |
|  |  | Sex, 16 males, 38 female |  |  |
|  |  | Exclusion: ill health, hospitalization in last 6 months | Setting: single centre |  |
|  |  | History: newly admitted |  |  |
| Tu 2004 [94] | 1. Loxapine dose mean 113 mg/day. *N* = 126 | Diagnosis: schizophrenia (CCMD-3) | Allocation: randomized | • CGI |
|  |  |  |  | • BPRS |
|  |  |  | Blinding: not mentioned | • Laboratory tests: ECG, bloods |
|  | 2. Chlorpromazine dose mean 428 mg/day. *N* = 112 | *N* = 238 |  |  |
|  |  |  | Duration 8 weeks |  |
|  |  | Age: adults, mean ~32 years, range 21–43 |  |  |
|  |  |  | Setting: multicenter |  |
|  |  | Sex 130 males, 108 females |  |  |
|  |  | History: hospitalized |  |  |
| Tuason 1984 [95] | 1. Loxapine dose range 30–150 mg/day. *N* = 34 | Diagnosis: schizophrenia, paranoid (RDC) | Allocation: randomized | • Leaving the study early |
|  |  |  |  | Note: Loss to follow-up 70% |
|  |  |  | Blinding: double—identical capsules |  |
|  |  |  |  | Only data from the outcome ‘leaving the study early’ included. |
|  | 2. Chlorpromazine dose range 300–1500 mg/day. *N* = 34 | *N* = 68 |  |  |
|  |  | Age: mean ~ 35 years, range 19–61 years | Duration 4 weeks—preceded by 8-h drug-free period |  |
|  |  |  |  | People who had improved were discharged and not followed up—adds to dropout over the 50% cut-off point |
|  | Doses individually titrated, antiparkinsonian and sedative medication as required |  |  |  |
|  |  | Sex: 32 males, 36 females |  |  |
|  |  |  | Setting: single centre |  |
|  |  | Exclusion: pregnancy or the risk of ill health, recent amphetamine abuse |  |  |
|  |  | History: mainly people with acute exacerbation of chronic illness, ill <1 week to >6 months to 20 years |  |  |
| Tuason 1986 [50] | 1. Loxapine: dose 25 mg IM, then 12.5–25 mg/h IM, max. 250 mg/day. *N* = 25 | Diagnosis: schizophrenia, acutely psychotic (DSM-III used beyond 3 days) | Allocation: randomized  Blinding: “modified double”—staff administering drugs not blinded, assessments blind | • General effect (requiring extended period of medication >24 h) |
|  | 2. Haloperidol: dose 5 mg IM, then 2.5–5 mg/h IM, max. 100 mg/day. *N* = 29 | *N* = 54 |  | • BPRS |
|  |  |  |  | • Side effects (sedation—ESBE, use of antiparkinsonian drugs) |
|  |  | Age: mean ~35 years (SD ~ 10), range 18–65 years |  |  |
|  |  |  |  | • Dropped from analysis |
|  |  |  |  | • Leaving the study early |
|  |  | Sex, 33 males, 19 females, 2 not reported |  |  |
|  |  | History: newly admitted |  |  |
|  |  |  | Duration: 24–72 h (IM phase)—oral phase data not included |  |
|  | Antiparkinsonian drugs and chloral hydrate as required |  |  |  |
|  |  |  | Setting: single centre |  |
|  |  | Inclusion criteria: >7 on BPRS hostility and uncooperativeness, behaviour = hostile/aggressive/uncooperative/unmanageable |  |  |
|  |  | Exclusion: ill health, co-existing mental illness condition |  |  |
| Van Der Velde 1975 [96] | 1. Loxapine: dose range 50–150 mg/day. *N* = 26 | Diagnosis: schizophrenia, acute or acute exacerbation (by two psychiatrists and principal investigator) | Allocation: randomized | • Dropped from analysis |
|  |  |  | Blinding: double—identical capsules | • Leaving the study early |
|  | 2. Thiothixene: dose range 20–60 mg/day. *N* = 28 |  |  |  |
|  |  |  |  | • Adverse effects |
|  |  |  | Duration: 6 weeks—preceded by 14-day drug-free period | • Laboratory tests |
|  | 3. Placebo. *N* = 28 |  |  |  |
|  |  | *N* = 82 |  |  |
|  |  | Age: mean ~ 27 years |  |  |
|  |  |  | Setting: single centre |  |
|  | Antiparkinsonian drugs and chloral hydrate as required |  |  |  |
|  |  | Sex, 43 males, 33 females, 6 not reported |  |  |
|  |  | Exclusion: not completing 2 weeks of study medication |  |  |
|  |  | History: 18/82 first episode, rest onset in last 6 years |  |  |
| Vyas 1980 [97] | 1. Loxapine: dose mean 44 mg/day, range 30–90 mg/day. *N* = 15 | Diagnosis: schizophrenia, chronic—no further details | Allocation: randomized | • CGI |
|  |  |  |  | • Leaving the study early |
|  |  |  | Blinding: double—identical capsules |  |
|  |  |  |  | • Adverse effects |
|  | 2. Chlorpromazine: dose mean 453 mg/day, range 300–900 mg/day. *N* = 15 | *N* = 30 |  |  |
|  |  | Age: mean ~ 32 years (SD ~ 9), all >21 | Duration 6 months—preceded by 15 day antipsychotic free period |  |
|  |  |  |  |  |
|  |  | Sex, 17 males, 13 females |  |  |
|  |  | Exclusion: pregnant women, ill health, substance abuse |  |  |
|  |  | History: inpatients, hospitalization duration <2 weeks to > 2 years |  |  |
|  |  |  | Setting: single centre |  |
|  | Antiparkinsonian drugs as required |  |  |  |
| Wang 1996 [98] | 1. Loxapine: dose range 50–300 mg/day. *N* = 30 | Diagnosis: schizophrenia (CCMD-2) | Allocation: randomized | • CGI |
|  |  |  |  | • BPRS |
|  |  |  |  | • TESS |
|  |  |  | Blinding: double blind | Physiological measures: temperature, BP, weight |
|  | 2. Chlorpromazine: dose range 75–600 mg/day. *N* = 30 | *N* = 60 |  |  |
|  |  | Age: mean ~36 years, range 18–60 | Duration 8 weeks |  |
|  |  |  | Setting: single centre |  |
|  |  | Sex, male and female—no further details |  |  |
|  |  | History: hospitalized |  |  |
| Wang 2005a [99] | 1. Loxapine: dose mean 267 mg/day. *N* = 34 | Diagnosis: schizophrenia | Allocation: randomized | • PANSS |
|  |  | *N* = 68 | Blinding: not mentioned |  |
|  | 2. Quetiapine: dose mean 426 mg/day. *N* = 34 | Age: mean ~33 years |  |  |
|  |  |  | Duration 8 weeks |  |
|  |  | Sex 32 males, 36 females |  |  |
|  |  | History: duration ill 3–12 months | Setting: single centre |  |
| Wang 2005b [100] | 1. Loxapine: dose mean 86 mg/day. *N* = 32 | Diagnosis: schizophrenia (CCMD-3) | Allocation: randomized  Blinding: not mentioned | • PANSS |
|  |  |  |  | • TESS |
|  |  |  |  | • Laboratory tests: ECG |
|  | 2. Risperidone: dose mean 4 mg/day. *N* = 31 | *N* = 63 |  |  |
|  |  | Age: mean ~29 years | Duration 8 weeks |  |
|  |  | Sex, 36 males, 30 females | Setting: single centre |  |
|  |  | History: hospitalized |  |  |
| Xue 2004 [101] | 1. Loxapine: dose range 34–136 mg/day. *N* = 100 | Diagnosis: schizophrenia (CCMD-3) | Allocation: randomized | • BPRS |
|  |  |  |  | • TESS |
|  |  |  | Blinding: double blind |  |
|  | 2. Chlorpromazine: dose range 250–500 mg/day. *N* = 100 | *N* = 200 |  |  |
|  |  |  | Duration: 8 weeks |  |
|  |  | Age: mean ~32 years |  |  |
|  |  | Sex 90 males, 110 females |  |  |
|  |  | History: in community |  |  |
| Zhang 2005 [102] | 1. Loxapine: dose range 34–68 mg/day. *N* = 44 | Diagnosis: schizophrenia (CCMD3) | Allocation: randomized | • PANSS |
|  |  |  | Blinding: not mentioned |  |
|  | 2. Perphenazine: dose range 6–12 mg/day. *N* = 46 | *N* = 134 |  |  |
|  |  |  | Duration 8 weeks |  |
|  |  | Age: mean ~30, range 18–60 |  |  |
|  |  | Sex: not reported | Setting: single centre |  |
|  | 3. Sulpiride: dose range 300–400 mg/day. *N* = 44 | History: hospitalized, mean duration ill ~4 years |  |  |

*Abbreviations*: PANSS-EC: Positive and negative syndrome scale excited component; CGI-I: Clinical Global Impression; ICD: International Classification of Disease; BPRS: Brief psychiatric rating scale; NOSIE: Nurses’ observation scale for inpatient evaluation; TESS: Toxic exposure surveillance system; DSM: Diagnostic and statistical manual of mental disorders; CCMD-3: Chinese classification of mental disorders 3; DOTES: Dosage record and treatment emergent symptom scale; EPSE: Extra pyramidal side effects; ECT: Electro-convulsive therapy; RDC: Research diagnostic criteria; SPS Slow progressive schizophrenia.
